# Supplementary material for: Structural Protein Effects Underpinning Cognitive Developmental Delay of the PURA p.Phe233del Mutation Modelled by Artificial Intelligence and the Hybrid Quantum Mechanics–Molecular Mechanics Framework
Source: Brain Sci. 2022 Jun 30;12(7):871. doi: 10.3390/brainsci12070871 (PMC9313109; doi:10.3390/brainsci12070871)
Supplement: Supplementary file 1 [file brainsci-12-00871-s001.zip › brainsci-1756739-supplementary.pdf]

brain sciences

MDPI

Article

Structural Protein Effects Underpinning Cognitive Developmental Delay of the *PURA* p.Phe233del Mutation Modelled by Artificial Intelligence and the Hybrid Quantum Mechanics–Molecular Mechanics Framework

Juan Javier López-Rivera [1,2,*], Luna Rodríguez-Salazar [3], Alejandro Soto-Ospina [4], Carlos Estrada-Serrato [2], David Serrano [2], Henry Mauricio Chaparro-Solano [2], Olga Londoño [2], Paula A. Rueda [3], Geraldine Ardila [3], Andrés Villegas-Lanau [4,5], Marcela Godoy-Corredor [6], Mauricio Cuartas [7], Jorge I. Vélez [8], Oscar M. Vidal [8], Mario A. Isaza-Ruget [1] and Mauricio Arcos-Burgos [9,*]

1 INPAC Research Group, Fundación Universitaria Sanitas, Bogotá 111321, Colombia
2 Grupo de Genética Médica, Clínica Universitaria Colombia y Clínica Pediátrica Colsanitas, Bogotá 111321, Colombia
3 Grupo de Bioinformática, Laboratorio de Clínica Colsanitas, Bogotá 110221, Colombia
4 Genética Molecular (GenMol), Facultad de Ciencias Exactas y Naturales, Universidad de Antioquia, Medellín 050012, Colombia
5 Grupo de Neurociencias de Antioquia (GNA), Facultad de Medicina, Universidad de Antioquia, Medellín 050012, Colombia
6 Laboratorio Clínico y de Patología, Clínica Colsanitas, Bogotá 110221, Colombia
7 Grupo de Investigación Estudios en Psicología, Departamento de Psicología, Escuela de Humanidades, Universidad EAFIT, Medellín 050022, Colombia
8 Universidad del Norte, Barranquilla 080001, Colombia
9 Grupo de Investigación en Psiquiatría (GIPSI), Departamento de Psiquiatría, Instituto de Investigaciones Médicas, Facultad de Medicina, Universidad de Antioquia, Medellín 050012, Colombia
* Correspondence: jjlopez@colsanitas.com (J.J.L.-R.); mauricio.arcos@udea.edu.co (M.A.-B.)

check for updates

Citation: López-Rivera, J.J.; Rodríguez-Salazar, L.; Soto-Ospina, A.; Estrada-Serrato, C.; Serrano, D.; Chaparro-Solano, H.M.; Londoño, O.; Rueda, P.A.; Ardila, G.; Villegas-Lanau, A.; et al. Structural Protein Effects Underpinning Cognitive Developmental Delay of the *PURA* p.Phe233del Mutation Modelled by Artificial Intelligence and the Hybrid Quantum Mechanics–Molecular Mechanics Framework. *Brain Sci.* **2022**, *12*, 871. https://doi.org/10.3390/brainsci12070871

Academic Editors: Andrew Chih Wei Huang, Seong Soo A. An, Bai Chuang Shyu, Muh-Shi Lin and Anna Kozłowska

Received: 19 May 2022
Accepted: 24 June 2022
Published: 30 June 2022

Publisher's Note: MDPI stays neutral with regard to jurisdictional claims in published maps and institutional affiliations.

Copyright: © 2022 by the authors. Licensee MDPI, Basel, Switzerland. This article is an open access article distributed under the terms and conditions of the Creative Commons Attribution (CC BY) license (https://creativecommons.org/licenses/by/4.0/).

Supplementary Figures

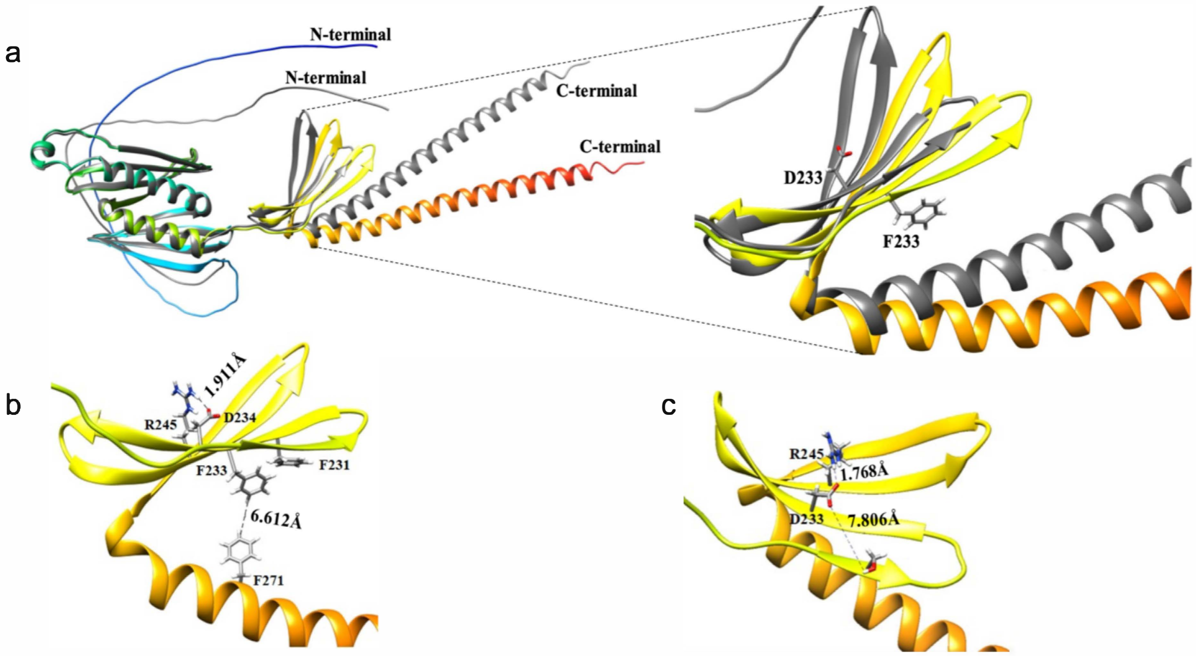

Figure S1. 3D analysis of PURAwt and PURA-Phe233del. **(a)** Needleman Wunsch global alignment for PURAwt (Rainbow) and PURA-Phe233del (Gray); **(b)** PURA wt protein region at 233; **(c)** PURA Phe233del protein region.

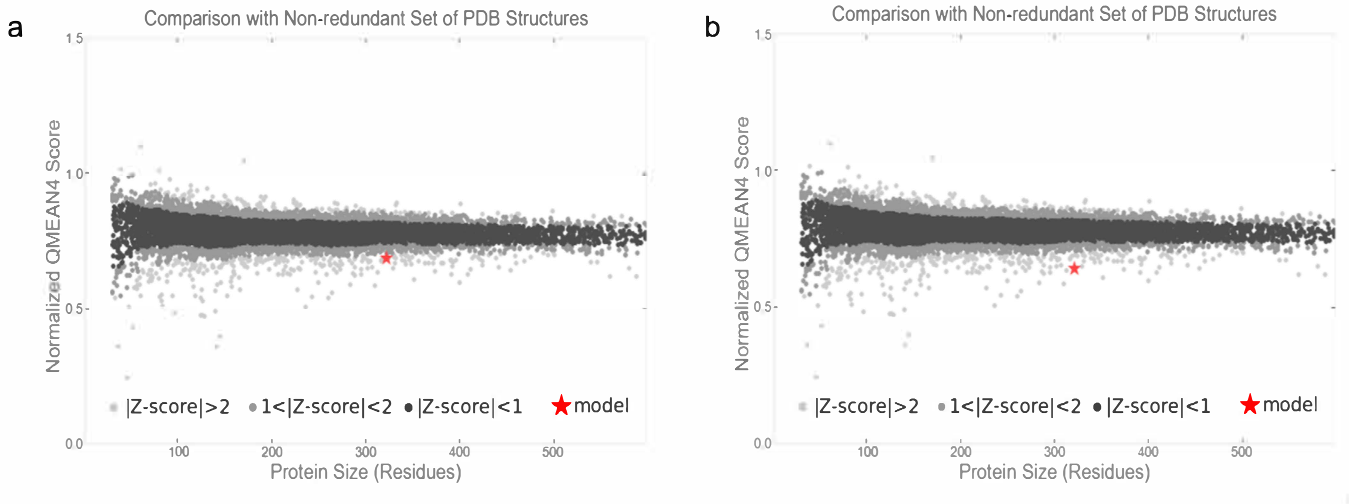

Figure S2. *Z*-score comparison diagram of experimentally solved structures for the **(a)** PURA wild type and **(b)** PURA Phe233del.

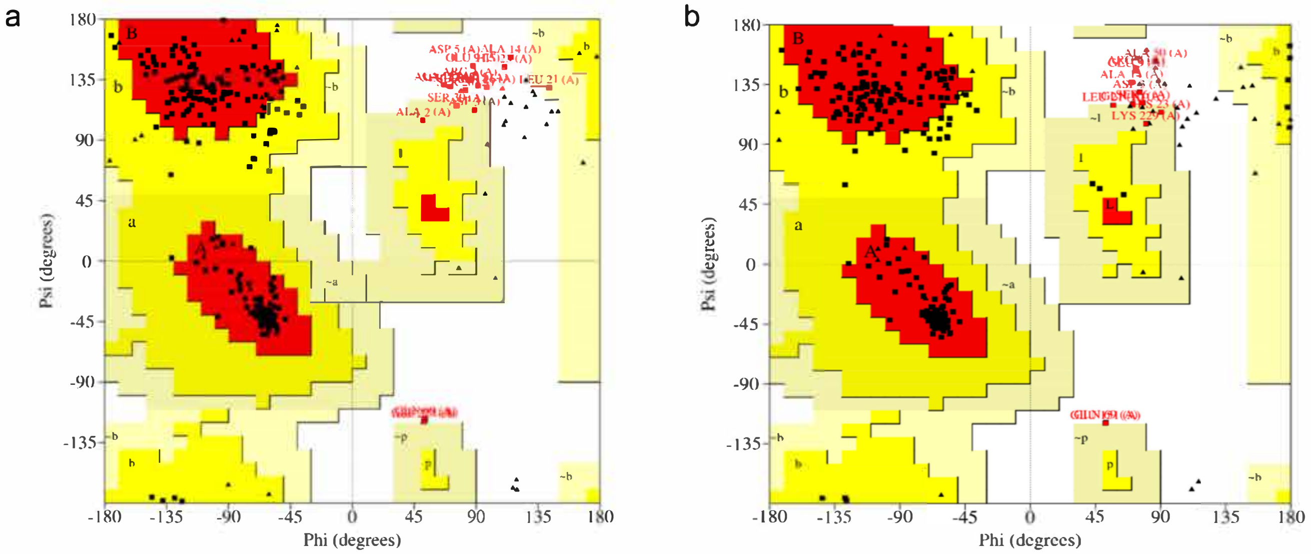

Figure S3. Ramachandran plots for the **(a)** PURAwt and **(b)** PURA-Phe233del using Procheck software.

Supplementary Tables

Table S1. NGS quality parameters.

Predictor DreamGenigs/ACMG Classification	Pathogenic
Gene	*PURA*
Chr	5
Coordinate	139494455
Ref	GCTT
Alt	G
c.Hgvs	c.697_699del
p.Hgvs	p.Phe233del
Protein Effect	INFRAME
Transcrito	NM_005859.5
Region	EXON
Zygosity/Father/Mother/Daugther	HOMZ_REF ǀ HOMZ_REF ǀ HETZ_ALT
Inheritance	AD
Coverage/Father/Mother/Daughter	165 ǀ 145 ǀ 162
Frequency/Alternate Allele/Father/Mother/Daughter	0 ǀ 0 ǀ 0.45679
Alt Count/Father/Mother/Daughter	0 ǀ 0 ǀ 74
ID dbSNP	rs786204835
Max Allele Freq	-
Max Allele Freq Origin	-
ACMG/Criteria	PS2+PM2+PP5
Phenotype OMIM/Additional, Information	Mental retardation, autosomal dominant 31 /
MIM Number	600473

HPO Terms	Delayed myelination / Absent speech / Feeding difficulties / Intellectual disability / Open mouth / Seizures / CNS hypomyelination / Autosomal dominant inheritance / Strabismus / Neonatal hypotonia / Myopathic facies / Respiratory insufficiency / Global developmental delay / Variable expressivity / Prominent forehead / Myoclonus / Broad-based gait / High palate / Telecanthus / Nystagmus

Table S2. Energetic and stereochemical characterization of the models.

Model	Software	QMEAN6	*Z*-score	Errat	Ramachandran Plot
PURA wt	AlphaFold2	0.61	-2.41	93.89	Favoured region: 219 (84.6%)
					Allowed region: 23 (8.9%)
					Generously allowed region: 4 (1.5%)
					Disallowed región: 13 (5.0%)
PURA Phe293del	AlphaFold2	0.59	-3.60	89.66	Favoured region: 216 (83.7%)
					Allowed region: 30 (11.6%)
					Generously allowed region: 3 (1.2%)
					Disallowed región: 9 (3.5%)
